# The vitamin D receptor is essential for the replication of pseudorabies virus

**DOI:** 10.1128/mbio.02137-24

**Published:** 2024-10-30

**Authors:** Lei Zeng, Shu-Yi Wang, Meng-Hua Du, Bei-Bei Chu, Sheng-Li Ming

**Affiliations:** 1 College of Veterinary Medicine, Henan Agricultural University, Zhengzhou, Henan, China; 2 Key Laboratory of Animal Biochemistry and Nutrition, Ministry of Agriculture and Rural Affairs, Zhengzhou, Henan, China; 3 Key Laboratory of Veterinary Biotechnology of Henan Province, Henan Agricultural University, Zhengzhou, Henan, China; 4 Longhu Advanced Immunization Laboratory, Zhengzhou, Henan, China; 5 International Joint Research Center of National Animal Immunology, Henan Agricultural University, Zhengzhou, Henan, China; 6 Ministry of Education Key Laboratory for Animal Pathogens and Biosafety, Zhengzhou, Henan, China; Columbia University Medical Center, New York, New York, USA

**Keywords:** PRV, VDR, p53, Ca^2+^, PI3K/AKT/mTORC1, AMPK/mTORC1, lipid synthesis

## Abstract

The vitamin D receptor (VDR) is a nuclear steroid receptor that regulates the expression of genes across various biological functions. However, the role of VDR in pseudorabies virus (PRV) infection has not yet been explored. We discovered that VDR positively influenced PRV proliferation because knockdown of VDR impaired PRV proliferation, whereas its overexpression promoted it. Additionally, we observed that PRV infection upregulated VDR transcription alongside 1,25-dihydroxyvitamin D3 (VD_3_) synthesis, contingent on p53 activation. Furthermore, VDR knockdown hindered PRV-induced lipid synthesis, implicating VDR’s involvement in this process. To decipher the mechanism behind VDR’s stimulation of lipid synthesis during PRV infection, we conducted RNA sequencing (RNA-seq) and found significant enrichment of genes in the Ca^2+^ signaling pathway. Measurements of Ca^2+^ indicated that VDR facilitated Ca^2+^ absorption. Moreover, the PI3K/AKT/mTORC1 and AMPK/mTORC1 pathways were also enriched in our RNA-seq data. Interfering with VDR expression, or chelating Ca^2+^ using BAPTA-AM, markedly impacted the activation of PI3K/AKT/mTORC1 and AMPK/mTORC1 pathways, lipid synthesis, and PRV proliferation. In summary, our study demonstrates that PRV infection promotes VDR expression, thereby enhancing Ca^2+^ absorption and activating PI3K/AKT/mTORC1- and AMPK/mTORC1-mediated lipid synthesis. Our findings offer new insights into strategies for PRV prevention.

**IMPORTANCE:**

Vitamin D, beyond its well-known benefits for bone health and immune function, also plays a pivotal role in regulating gene expression through its receptor, the vitamin D receptor (VDR). Although VDR’s influence spans multiple biological processes, its relationship with viral infections, particularly pseudorabies virus (PRV), remains underexplored. Our research illustrates a complex interplay where PRV infection boosts VDR expression, which in turn enhances Ca^2+^ absorption, leading to the activation of critical lipid synthesis pathways, PI3K/AKT/mTORC1 and AMPK/mTORC1. These findings not only deepen our understanding of the intricate dynamics between host molecular mechanisms and viral proliferation but also open avenues for exploring new strategies aimed at preventing PRV infection. By targeting components of the VDR-related signaling pathways, we can potentially develop novel therapeutic interventions against PRV and possibly other similar viral infections.

## INTRODUCTION

Pseudorabies virus (PRV), also known as Aujeszky’s disease or mad itch, predominantly affects swine but can also infect a wide range of other animals such as cattle, sheep, goats, cats, dogs, and wild rodents (1, 2). PRV belongs to the Herpesviridae family and can cause neurological disorders, respiratory distress, and reproductive issues among the infected populace (3). Importantly, although PRV poses no direct threat to human health, it significantly impacts animal welfare and the swine industry, making it a pressing concern in veterinary medicine and agricultural economics (3, 4).

The vitamin D receptor (VDR) is a cellular protein, which, in conjunction with vitamin D3 (VD_3_), plays a crucial role in the transcription of genetic information from DNA to messenger RNA, thus regulating cell growth, immune function, and other vital physiological processes (5, 6). Emerging research has unveiled the significant influence of VDR on the dynamics between viruses and their hosts. Both VD_3_ and its receptor are being explored as potential therapeutic agents for viral infections, given their ability to foster an antiviral state within host cells, which helps regulate and curb viral replication (7, 8). Specifically, VDR has been implicated in enhancing the immune response to viruses such as influenza and human immunodeficiency virus (HIV) (9). There is also preliminary evidence suggesting VDR’s involvement in the body’s defense against the SARS-CoV-2 virus responsible for COVID-19 (10). These insights underscore the necessity for further investigation into how VD_3_ supplementation could potentially serve as a preventative or therapeutic measure against viral infections. The well-established roles of VD_3_ and VDR in immune modulation further highlight the importance of maintaining optimal VD_3_ levels for general health and infection resistance.

Recently, the intricate relationship between lipid metabolism and viral infections has gained considerable attention (11). Lipid metabolism, the process of breaking down or synthesizing lipids for energy production and cellular activities, becomes critically altered during viral infections (12). Viruses, as obligate intracellular parasites, modify host lipid metabolism to suit their replication, assembly, and egress, essentially commandeering cellular machinery to produce lipid profiles that favor their lifecycle and modulate host immune responses (13, 14). Notably, the Hepatitis C virus manipulates liver lipid metabolism to foster a lipid-rich environment conducive to its replication (15). Similarly, HIV disrupts lipid metabolism to facilitate the synthesis and storage of lipids, thereby supporting the generation of new virions (16). Understanding the link between lipid metabolism and viral infections not only enhances our knowledge of virus-host interactions but also opens new avenues for potential antiviral therapies.

In this study, we shed light on the mechanism by which PRV infection activates the VD_3_/VDR signaling pathway, inducing an imbalance in Ca^2+^ levels that, in turn, stimulates lipid synthesis via the PI3K/AKT/mTORC1 and AMPK/mTORC1 pathways, thereby promoting viral replication. This work not only contributes to our understanding of the complex interactions between host signaling pathways and viral lifecycle but also points to novel therapeutic targets for controlling PRV infection.

## RESULTS

### VDR is involved in and is upregulated by PRV infection

**Fig 1:**
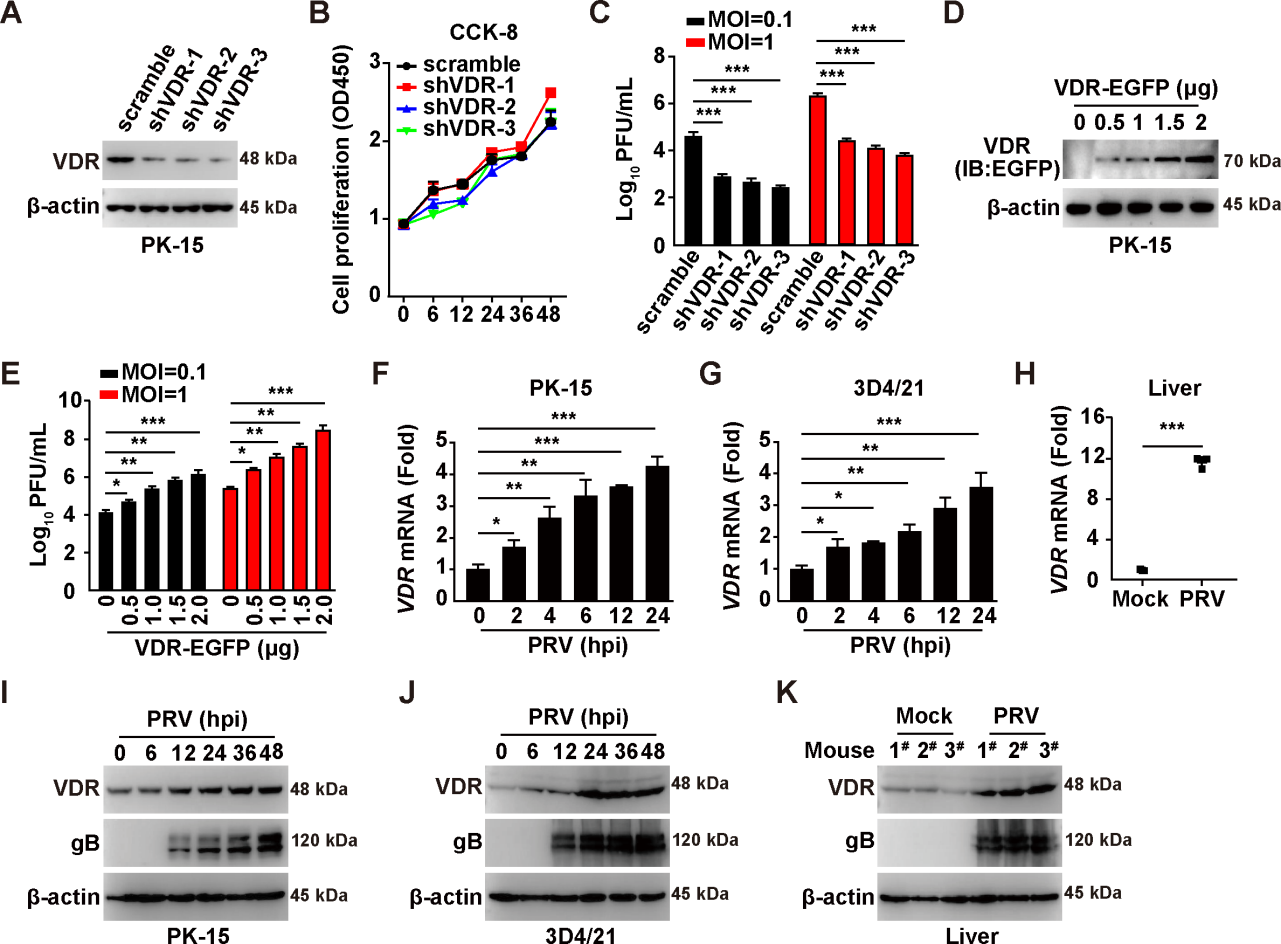
PRV infection upregulates VDR expression to promote viral proliferation. (**A**) The protein levels of VDR in scramble, shVDR-1, shVDR-2, and shVDR-3 PK-15 cells were analyzed by immunoblotting analysis. (**B**) Cell proliferation was analyzed in scramble, shVDR-1, shVDR-2, and shVDR-3 PK-15 cells for 0–48 h by CCK-8 assay. (**C**) Scramble, shVDR-1, shVDR-2, and shVDR-3 PK-15 cells were infected with PRV-QXX (MOI = 0.1 and 1) for 24 h. The viral titer was analyzed by a PFU assay. ^***^P < 0.001. (**D**) PK-15 cells were transfected with VDR-EGFP plasmid (0–2 µg) for 24 h. VDR-EGFP expression was analyzed by immunoblotting analysis. (**E**) PK-15 cells were transfected with VDR-EGFP plasmid (0–2 µg) for 24 h. Cells were then infected with PRV-QXX (MOI = 0.1 and 1) for another 24 h. The viral titer was analyzed by a PFU assay. ^*^P < 0.05, ^**^P < 0.01, ^***^P < 0.001. (**F and G**) PK-15 (**F**) and 3D4/21 (**G**) cells were infected with PRV-QXX (MOI = 1) for 0–24 h. The mRNA levels of VDR were analyzed using qRT-PCR analysis. ^*^P < 0.05, ^**^P < 0.01, ^***^P < 0.001. (**H**) C57BL/6 J mice were mock‐infected or intranasally infected with PRV-QXX (5 × 10^3^ TCID_50_ per mouse) for 3 days. The mRNA levels of VDR in murine liver were analyzed by qRT-PCR analysis (*n* = 3). ^***^P < 0.001. (**I and J**) PK-15 (**I**) and 3D4/21 (**J**) cells were infected with PRV-QXX (MOI = 0.1) for 0–48 h. VDR, gB, and β-actin were analyzed by immunoblotting analysis. (**K**) The protein levels of VDR, gB, and β-actin were analyzed by immunoblotting analysis in murine liver from (**H**).

To elucidate the role of VDR in PRV infection, we employed a combination of RNA interference (RNAi) and overexpression strategies. Specifically, we designed three short hairpin RNAs (shRNAs) targeting porcine VDR mRNA. These shRNAs were then packaged into lentiviruses and used to infect PK-15 cells. Immunoblotting analysis revealed that VDR expression was significantly reduced by these three shRNAs (Fig. 1A). Subsequently, we assessed the impact of VDR knockdown on cell proliferation using the Cell Counting Kit-8 (CCK-8) assay. Over a 48-h cultivation period, we observed no noticeable difference in the proliferation rates of control cells compared with those with VDR knockdown (Fig. 1B). However, plaque assays conducted with PRV-QXX at different multiplicities of infection (MOI of 0.1 and 1) suggested that suppression of VDR expression detrimentally affected PRV proliferation (Fig. 1C). In contrast, we achieved VDR overexpression by transfecting PK-15 cells with a plasmid encoding VDR-EGFP (Fig. 1D). Cells overexpressing VDR yielded a higher number of PRV progeny viruses compared with cells transfected with the control vector (Fig. 1E), indicating a positive regulation of PRV proliferation by VDR.

To further investigate the relationship between PRV infection and VDR expression, we infected PK-15, 3D4/21 cells, and mice with PRV-QXX and analyzed VDR transcription. Quantitative real-time polymerase chain reaction (qRT-PCR) analysis revealed significant inductions of VDR mRNA in both PK-15 cells and murine liver following PRV infection (Fig. 1F through H). We also examined the protein levels of VDR during PRV infection through immunoblotting analysis, which showed increased VDR expression in cell cultures and murine liver post-infection (Fig. 1I through K). These findings indicate that PRV infection stimulates VDR expression to facilitate viral proliferation.

### PRV-induced p53 activation is responsible for VDR expression

**Fig 2:**
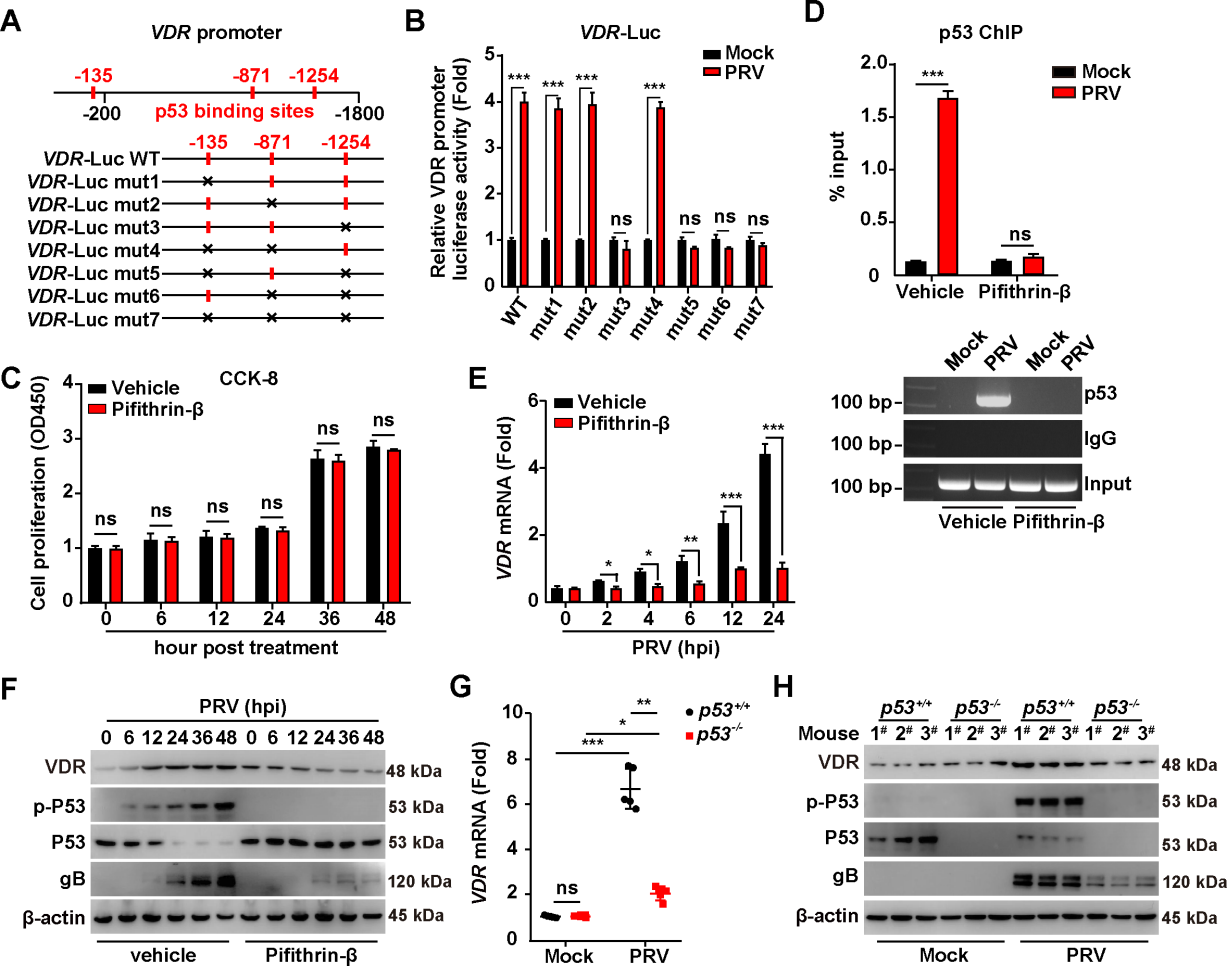
PRV infection triggers VDR expression through p53 activation. (**A**) Schematic diagram of the VDR promoter with potential p53 binding sites and the various mutant constructions. (**B**) PK-15 cells were transfected with the indicated VDR-LUC plasmids for 12 h and then mock-infected or infected with PRV-QXX (MOI = 1) for 24 h. VDR promoter activities were analyzed by dual-luciferase reporter assay. ***P < 0.001. ns, no significance. (**C**) PK-15 cells were treated with vehicle or Pifithrin-β (10 µM) for 0–48 h. Cell proliferation was analyzed by CCK-8 assay. ns, no significance. (**D**) PK-15 cells were treated with vehicle or Pifithrin-β (10 µM) and simultaneously mock-infected or infected with PRV-QXX (MOI = 1) for 24 h. The interaction of p53 with the VDR promoter was analyzed using a p53 ChIP assay. ***P < 0.001. ns, no significance. (**E**) PK-15 cells were treated with vehicle or Pifithrin-β (10 µM) and simultaneously infected with PRV-QXX (MOI = 1) for 0–24 h. The mRNA levels of VDR were analyzed using qRT-PCR analysis. ^*^P < 0.05, ^**^P < 0.01, ^***^P < 0.001. (**F**) PK-15 cells were treated with vehicle or Pifithrin-β (10 µM) and simultaneously infected with PRV-QXX (MOI = 0.1) for 0–48 h. The protein levels of VDR, p-p53, p53, gB, and β-actin were analyzed by immunoblotting analysis. (**G**) *p53^+/+^* and *p53^-/^*^-^ mice were mock-infected or intranasally infected with PRV-QXX (5 × 10^3^ TCID_50_ per mouse) for 3 days. The mRNA levels of VDR in murine liver were analyzed by qRT-PCR analysis (*n* = 5). ^*^P < 0.05, ^**^P < 0.01, ^***^P < 0.001. ns, no significance. (**H**) The protein levels of VDR, p-p53, p53, gB, and β-actin were analyzed by immunoblotting analysis in murine liver from (**G**).

We further explored the mechanism behind the transcriptional upregulation of VDR expression induced by PRV. Promoter analysis revealed three potent p53 binding sites within the VDR promoter (Fig. 2A). Previous reports have indicated that p53 is essential for the proliferation of herpes simplex virus 1 (17), prompting us to first identify the specific p53 binding sites crucial for PRV-induced VDR expression. Consequently, we generated seven mutations across the p53 binding sites in the VDR promoter (Fig. 2A). Dual luciferase reporter assay assessing VDR promoter activity revealed that mutation of the third p53 binding site (−1254) abrogated VDR promoter activation in response to PRV infection, whereas mutations in the first two binding sites (−135 and −871) did not (Fig. 2B). This prompted an investigation into the direct binding of p53 to the VDR promoter. Pifithrin-β is a compound known to antagonize p53 activity without influencing cell proliferation (Fig. 2C). Chromatin immunoprecipitation (ChIP) assays confirmed the recruitment of p53 to the VDR promoter during PRV infection (Fig. 2D). This recruitment was absent in Pifithrin-β-treated cells (Fig. 2D), implicating a pivotal role for p53 in mediating PRV-stimulated VDR transcription.

PRV failed to stimulate VDR transcription in cells treated with Pifithrin-β compared with vehicle-treated controls (Fig. 2E). Immunoblotting analysis corroborated that Pifithrin-β inhibited both the activation of p53 (via phosphorylation) and the expression of VDR during PRV infection (Fig. 2F). The essential role of p53 in modulating VDR expression *in vivo* was confirmed using p53-knockout mice. Both *p53^+/+^* and *p53^-/-^* mice were subjected to either mock infection or intranasal infection with PRV-QXX for 3 days. qRT-PCR analysis revealed an approximately 7-fold increase in VDR mRNA in the liver of PRV-infected *p53^+/+^* mice, as opposed to those mock-infected (Fig. 2G). This induction was markedly suppressed in the absence of *p53* (Fig. 2G). Likewise, immunoblotting analysis demonstrated that the absence of p53 inhibited the PRV-induced upregulation of VDR in the liver of infected mice (Fig. 2H). Together, these results demonstrate the crucial role of PRV-induced p53 activation in driving the expression of VDR.

### VD_3_ is critical for VDR-mediated PRV infection

**Fig 3:**
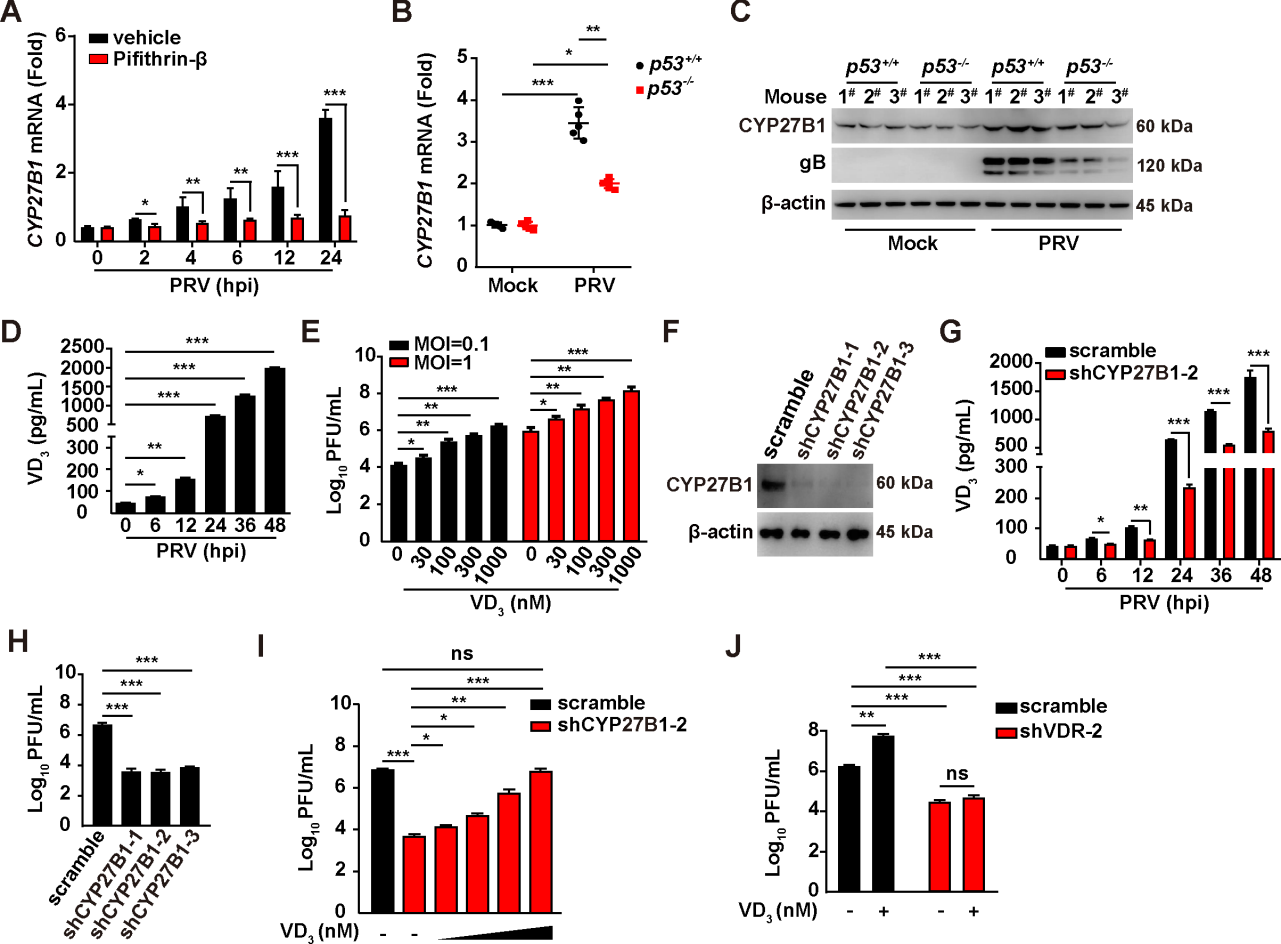
PRV infection activates VD_3_ synthesis to assist viral replication. (**A**) PK-15 cells were treated with vehicle or Pifithrin-β (10 µM) and simultaneously infected with PRV-QXX (MOI = 1) for 0–24 h. The mRNA levels of CYP27B1 were analyzed by qRT-PCR analysis. ^*^P < 0.05, ^**^P < 0.01, ^***^P < 0.001. (**B**) *p53^+/+^* and *p53^-/^*^-^ mice were mock-infected or intranasally infected with PRV-QXX (5 × 10^3^ TCID_50_ per mouse) for 3 days. The mRNA levels of CYP27B1 in murine liver were analyzed by qRT-PCR analysis (*n* = 5). ^*^P < 0.05, ^**^P < 0.01, ^***^P < 0.001. (**C**) *p53^+/+^* and *p53^-/^*^-^ mice were mock-infected or intranasally infected with PRV-QXX (5 × 10^3^ TCID_50_ per mouse) for 3 days. The protein levels of CYP27B1, gB, and β-actin in murine liver were analyzed by immunoblotting analysis. (**D**) PK-15 cells were infected with PRV-QXX (MOI = 0.1) for 0–48. Intracellular VD_3_ levels were analyzed by ELISA. ^*^P < 0.05, ^**^P < 0.01, ^***^P < 0.001. (**E**) PK-15 cells were treated with VD_3_ (0–1000 nM), and simultaneously infected with PRV-QXX (MOI = 0.1 and 1) for 24 h. The viral titer was analyzed by a PFU assay. ^*^P < 0.05, ^**^P < 0.01, ^***^P < 0.001. (**F**) The protein levels of CYP27B1 in scramble, shCYP27B1-1, shCYP27B1-2, and shCYP27B1-3 PK-15 cells were analyzed by immunoblotting analysis. (**G**) Scramble and shCYP27B1-2 PK-15 cells were infected with PRV-QXX (MOI = 0.1) for 0–48. Intracellular VD_3_ levels were analyzed by ELISA. ^*^P < 0.05, ^**^P < 0.01, ^***^P < 0.001. (**H**) Scramble, shCYP27B1-1, shCYP27B1-2, and shCYP27B1-3 PK-15 cells were infected with PRV-QXX (MOI = 1) for 24 h. The viral titer was analyzed by a PFU assay. ^***^P < 0.001. (**I**) Scramble and shCYP27B1-2 PK-15 cells were infected with PRV-QXX (MOI = 1) and treated with VD_3_ (0–1,000 nM) as indicated for 24 h. The viral titer was analyzed by a PFU assay. ^*^P < 0.05, ^**^P < 0.01, ^***^P < 0.001. ns, no significance. (**J**) Scramble and shVDR-2 PK-15 cells were infected with PRV-QXX (MOI = 1) and treated with VD_3_ (1000 nM) as indicated for 24 h. The viral titer was analyzed by a PFU assay. ^**^P < 0.01, ^***^P < 0.001. ns, no significance.

VD_3_ serves as the natural ligand for VDR and is produced by the enzyme CYP family 27 subfamily B member 1 (CYP27B1). To assess the importance of VD_3_ in VDR-mediated PRV infection, we first explored whether PRV infection influenced CYP27B1 expression. Intriguingly, PRV infection was observed to elevate CYP27B1 mRNA and protein levels in PK-15 cells and murine liver (Fig. 3A through C). However, this upregulation was mitigated upon the inhibition of p53, either through the usage of pifithrin-β or via the knockout of *p53* (Fig. 3A through C). This suggests a role for PRV-induced p53 activation in governing CYP27B1 expression. Subsequent analysis measured the synthesis of VD_3_ following PRV infection, revealing a significant increase in VD_3_ production, as indicated by enzyme-linked immunosorbent assay (ELISA) results (Fig. 3D). Moreover, the supplementation of VD_3_ in the culture medium markedly improved the yield of PRV progeny virus (Fig. 3E), highlighting the stimulatory effect of PRV infection on VD_3_ synthesis in facilitating viral replication.

To corroborate the significance of VD_3_ in the context of PRV replication, we employed an RNAi-mediated approach to knock down CYP27B1. Three shRNAs targeting porcine CYP27B1 mRNA were designed, packaged into lentiviruses, and used to infect PK-15 cells. Immunoblotting analysis confirmed that CYP27B1 expression was significantly diminished by these three shRNAs (Fig. 3F). ELISA assays further demonstrated that CYP27B1 knockdown led to a reduction in VD_3_ synthesis in the context of PRV infection (Fig. 3G). Correspondingly, the production of PRV progeny virus decreased in shCYP27B1 PK-15 cells infected with PRV compared with control cells (Fig. 3H). Notably, decreased PRV replication in shCYP27B1-2 PK-15 cells was reversed with VD_3_ treatment (Fig. 3I). To assess whether the VD_3_-promoted PRV proliferation depended on the presence of VDR, we supplemented the culture medium with VD_3_ in cells subjected to VDR knockdown. The results indicated that PRV proliferation decreased in shVDR-2 PK-15 cells treated with either vehicle or VD_3_, relative to control cells (Fig. 3J). Collectively, our findings strongly suggest that VD_3_ plays a vital role in facilitating VDR-mediated PRV replication.

### PRV infection results in increased cytoplasmic Ca^2+^ via VDR

**Fig 4:**
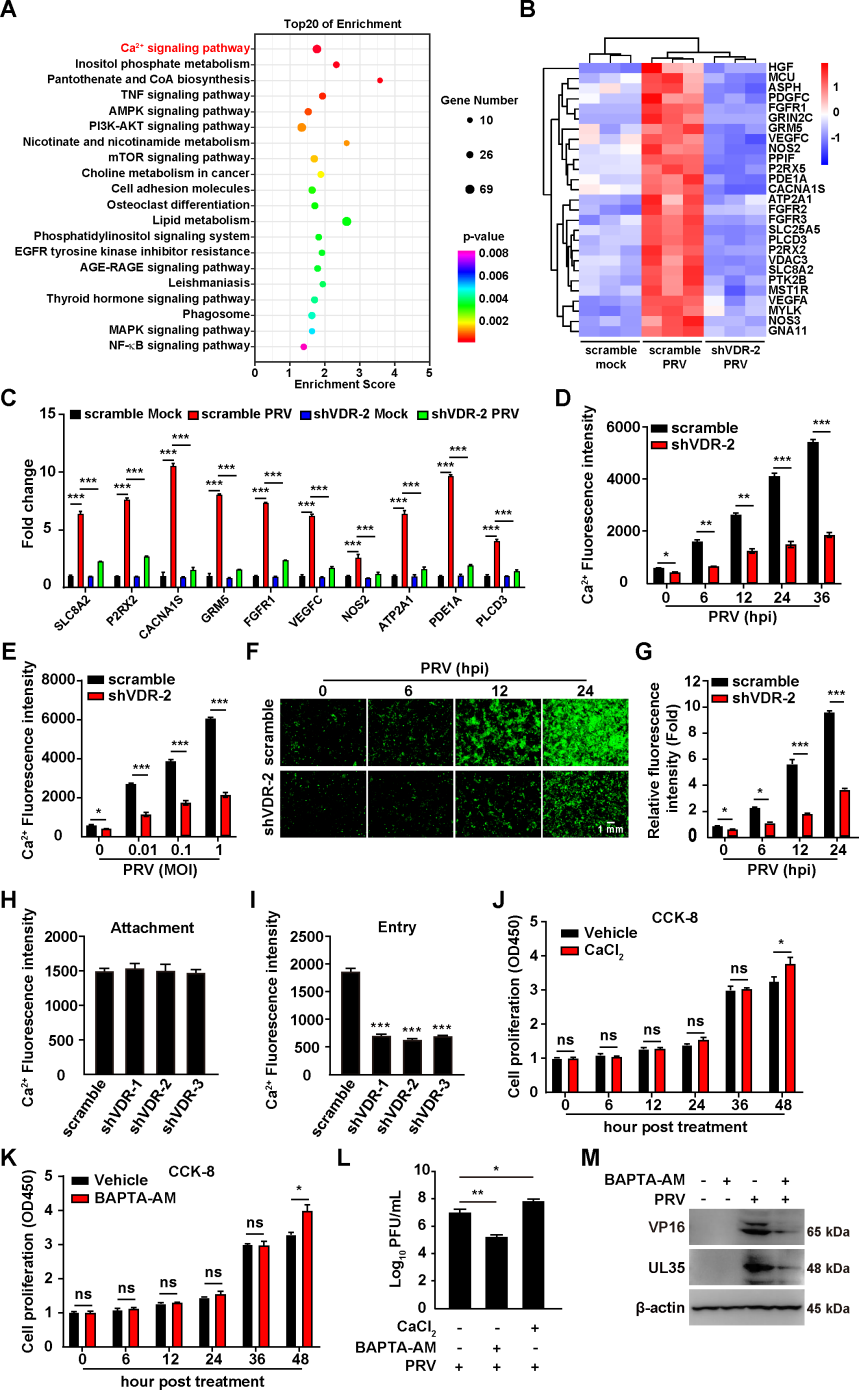
PRV infection promotes an increased cytoplasmic Ca^2+^ through VDR. (**A**) KEGG-enriched metabolic pathways in scramble and shVDR-2 PK-15 cells mock-infected or infected with PRV-QXX (MOI = 1) as indicated for 24 h. (**B**) Heat map of the fold changes of the indicated Ca^2+^ signaling pathway genes in scramble and shVDR-2 PK-15 cells mock-infected or infected with PRV-QXX (MOI = 1) as indicated for 24 h. (**C**) Scramble and shVDR-2 PK-15 cells were mock-infected or infected with PRV-QXX (MOI = 1) for 24 h. The mRNA levels of the indicated genes in Ca^2+^ signaling pathway were analyzed by qRT-PCR analysis. ^***^P < 0.001. (**D**) Scramble and shVDR-2 PK-15 cells were infected with PRV-QXX (MOI = 0.1) for 0–36 h. Intracellular Ca^2+^ was detected by Fluo-4 staining. ^*^P < 0.05, ^**^P < 0.01, ^***^P < 0.001. (**E**) Scramble and shVDR-2 PK-15 cells were infected with PRV-QXX (MOI = 0, 0.01, 0.1, 1) for 24 h. Intracellular Ca^2+^ was detected by Fluo-4 staining. ^*^P < 0.05, ^***^P < 0.001. (**F**) Scramble and shVDR-2 PK15 cells were infected with PRV-QXX (MOI = 1) for 0–24 h. Intracellular Ca^2+^ was detected by confocal microscopy with Fluo-4 staining. Scale bar, 1 mm. (**G**) Quantification of the relative Ca^2+^ fluorescence intensity from (**F**). ^*^P < 0.05, ^***^P < 0.001. (**H**) Scramble, shVDR-1, shVDR-2, and shVDR-3 PK-15 cells were incubated with PRV (MOI = 10) at 4°C for 10 min. Intracellular Ca^2+^ was detected by Fluo-4 staining. (**I**) Scramble, shVDR-1, shVDR-2, and shVDR-3 PK-15 cells were incubated with PRV (MOI = 10) at 4°C for 2 h, extensively washed with ice-cold PBS three times, and then incubated at 37°C for 10 min to allow entry. Intracellular Ca^2+^ was detected by Fluo-4 staining. ^***^P < 0.001. (**J and K**) PK-15 cells were treated with vehicle, CaCl_2_ (J, 10 µM), or BAPTA-AM (K, 10 µM) for 0–48 h. Cell proliferation was analyzed by CCK-8 assay. ^*^P < 0.05. ns, no significance. (**L**) PK-15 cells were treated with CaCl_2_ (10 µM) or BAPTA-AM (10 µM) as indicated and simultaneously infected with PRV-QXX (MOI = 1) for 0–24 h. The viral titer was analyzed by a PFU assay. ^*^P < 0.05, ^**^P < 0.01. (**M**) PK-15 cells were treated with BAPTA-AM (10 µM) and simultaneously mock-infected or infected with PRV-QXX (MOI = 1) as indicated for 24 h. The protein levels of PRV VP16, UL35, and β-actin were analyzed by immunoblotting analysis.

To investigate how VDR facilitated the proliferation of PRV, we conducted initial experiments with mock-infected scramble, PRV-infected scramble, and PRV-infected shVDR-2 PK-15 cells. Each condition was replicated three times biologically. RNA sequencing (RNA-seq) was performed on samples collected 24 h post-PRV infection. We then utilized the Kyoto Encyclopedia of Genes and Genomes (KEGG) for pathway analysis of the RNA-seq data to predict the biological processes involved. This revealed a significant enrichment of genes primarily involved in the Ca^2+^ signaling pathway (Fig. 4A). Specifically, 27 genes within this pathway were upregulated in control cells but showed decreased expression in VDR-knockdown cells under PRV infection conditions (Fig. 4B). To corroborate these findings, we performed qRT-PCR analysis on 10 of the 27 identified genes. As shown in Fig. 4C, PRV infection increased the mRNA levels of genes including SLC8A2, P2R × 2, CACNA1S, GRM5, FGFR1, VEGFC, NOS2, ATP2A1, PDE1A, and PLCD3 in control cells, whereas this upregulation was diminished by VDR knockdown, emphasizing VDR’s significant role in modifying the expression of these Ca^2+^ signaling pathway genes in response to PRV infection.

**Fig 5:**
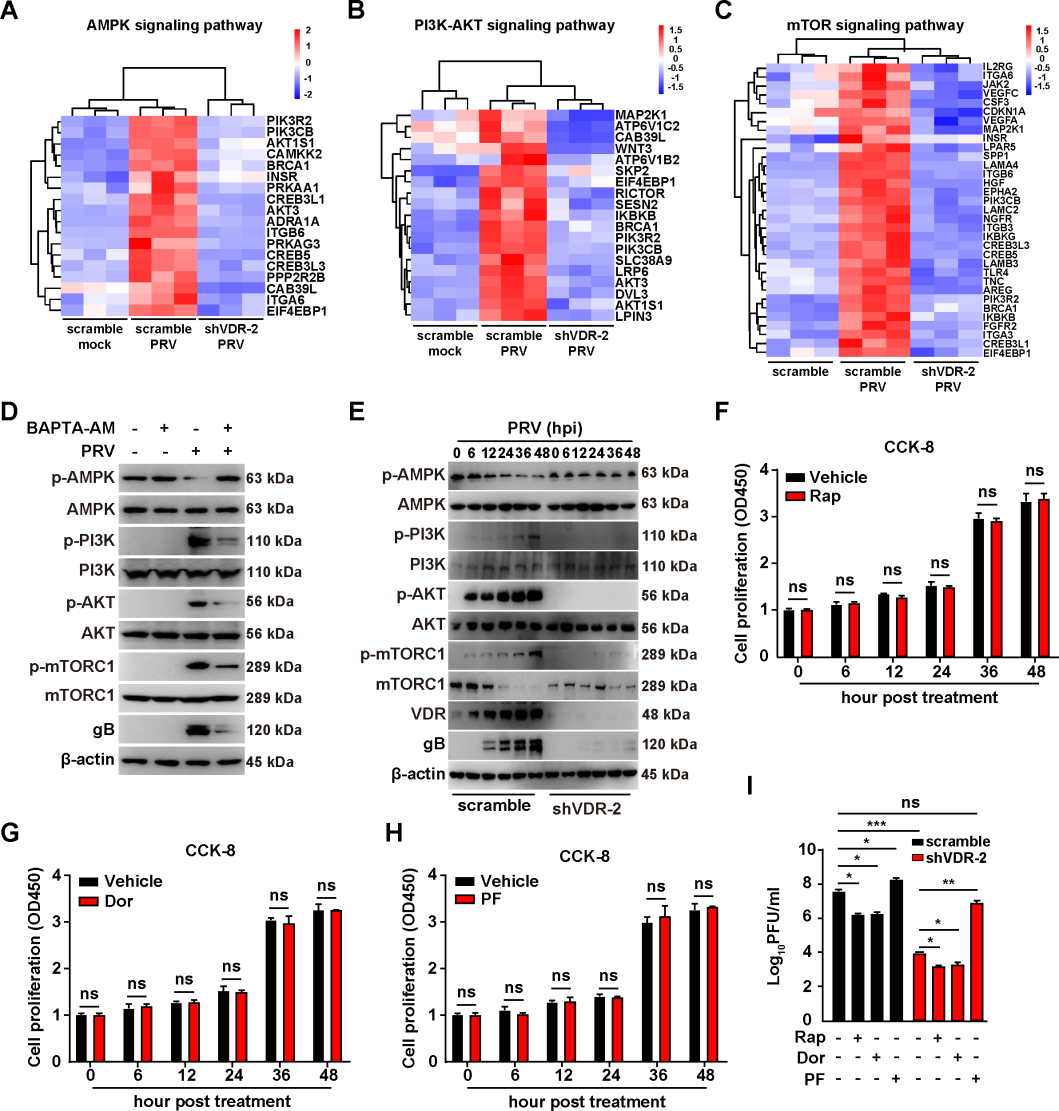
PRV infection activates The PI3K/AKT/mTORC1 and AMPK/mTORC1 pathways via VDR-mediated Ca^2+^ absorption. (**A**) Heat map of the fold changes of the indicated AMPK signaling pathway genes in scramble and shVDR-2 PK-15 cells mock-infected or infected with PRV-QXX (MOI = 1) as indicated for 24 h. (**B**) Heat map of the fold changes of the indicated PI3K-AKT signaling pathway genes in scramble and shVDR-2 PK-15 cells mock-infected or infected with PRV-QXX (MOI = 1) as indicated for 24 h. (**C**) Heat map of the fold changes of the indicated mTOR signaling pathway genes in scramble and shVDR-2 PK-15 cells mock-infected or infected with PRV-QXX (MOI = 1) as indicated for 24 h. (**D**) PK15 cells were treated with BAPTA-AM (10 µM) as indicated, and simultaneously mock-infected or infected with PRV-QXX (MOI = 1) for 24 h. The protein levels of p-AMPK, AMPK, p-PI3K, PI3K, p-AKT, AKT, p-mTORC1, mTORC1, PRV gB, and β-actin were analyzed by immunoblotting analysis. (**E**) Scramble and shVDR-2 PK15 cells were infected with RV-QXX (MOI = 0.1) for 0–48 h. The protein levels of p-AMPK, AMPK, p-PI3K, PI3K, p-AKT, AKT, p-mTORC1, mTORC1, VDR, PRV gB, and β-actin were analyzed by immunoblotting analysis. (**F-H**) PK-15 cells were treated with vehicle, Rap (F, 10 µM), Dor (G, 100 nM), or PF (H, 10 µM) for 0–48 h. Cell proliferation was analyzed by CCK-8 assay. ns, no significance. (**I**) Scramble and shVDR-2 PK15 cells were treated with Rap (10 µM), Dor (100 nM), and PF (10 µM) as indicated and infected with PRV-QXX (MOI = 1) for 24 h. The viral titer was analyzed by a PFU assay. ^*^P < 0.05, ^**^P < 0.01, ^***^P < 0.001. ns, no significance.

Deletion of VDR has previously been demonstrated to impair basal Ca^2+^ absorption, compromising mice’s ability to adjust to low-Ca^2+^ diets (18). To examine the potential effect of PRV infection on intracellular Ca^2+^ levels via VDR, we monitored intracellular Ca^2+^ dynamics in both scramble and shVDR-2 PK15 cells post-PRV infection. A time-dependent increase in cytoplasmic Ca^2+^ was documented in PRV-infected scramble cells, correlating with both extended infection periods and increased MOI (Fig. 4D and E). Furthermore, Ca^2+^ fluorescence intensity, visualized using Fluo-4 AM dye in green, intensified from 6 to 36 h post-infection (Fig. 4F and G), indicating an upsurge in intracellular Ca^2+^ levels. In contrast, shVDR-2 PK-15 cells exhibited reduced Fluo-4 fluorescence (Fig. 4F and G), suggestive of a diminished Ca^2+^ influx or retention.

Next, we examined Ca^2+^ absorption during the early stage of PRV infection. It was observed that reducing VDR levels during the PRV attachment phase did not affect Ca^2+^ absorption, whereas decreasing VDR during the entry phase significantly hindered cellular Ca^2+^ absorption (Fig. 4H and I). To delineate the role of Ca^2+^ in PRV propagation, we utilized BAPTA-AM, a Ca^2+^ chelator, to sequester intracellular Ca^2+^, and alternatively, enhanced extracellular Ca^2+^ using CaCl_2_. CCK-8 assay indicated that treatment of PK-15 cells with Ca^2+^ or BAPTA-AM did not influence cell proliferation before 48 h (Fig. 4J and K). Assessment of viral titers showed that CaCl_2_ supplementation promoted, whereas BAPTA-AM-mediated chelation impeded PRV progeny production (Fig. 4L). Immunoblotting analysis indicated that BAPTA-AM treatment inhibited the expression of PRV VP16 and UL35 (Fig. 4M). Taken together, these results indicate that VDR is crucial for regulating Ca^2+^ absorption triggered by PRV infection.

### VDR-mediated Ca^2+^ absorption is critical for the activation of PI3K/AKT/mTORC1 and AMPK/mTORC1 pathways during PRV infection

The enrichment analysis of the KEGG pathway revealed significant enrichment of parent genes predominantly in the PI3K/AKT/mTORC1 and AMPK/mTORC1 pathways (Fig. 4 and 5A through C), which are modulated by Ca^2+^ (19, 20). Our data demonstrated that PRV infection promoted VDR-dependent Ca^2+^ absorption (Fig. 4), leading to the hypothesis that VDR-mediated Ca^2+^ absorption played a crucial role in activating the PI3K/AKT/mTORC1 and AMPK/mTORC1 pathways. To explore this further, we treated cells with Ca^2+^ chelator BAPTA-AM and conducted immunoblotting analysis to assess the phosphorylation of AMPK, PI3K, AKT, and mTORC1. We observed an increase in phosphorylated AMPK (p-AMPK) in BAPTA-AM-treated cells compared with vehicle-treated cells following PRV infection (Fig. 5D), suggesting the involvement of Ca^2+^. In contrast, BAPTA-AM treatment resulted in decreased phosphorylation levels of PI3K (p-PI3K), AKT (p-AKT), and mTORC1 (p-mTORC1) in PRV-infected PK-15 cells (Fig. 5D), reinforcing the significance of VDR-mediated Ca^2+^ absorption in the activation of these pathways.

To further elucidate VDR’s role in this process, we analyzed cellular responses to PRV infection under normal conditions and VDR knockdown. In control cells, PRV infection led to decreased p-AMPK and increased p-PI3K, p-AKT, and p-mTORC1 levels, changes not observed in VDR-knockdown cells (Fig. 5E). These findings indicate that VDR was integral to the activation of the PI3K/AKT/mTORC1 and AMPK/mTORC1 pathways during PRV infection. Further exploration involved treating control and shVDR-2 PK-15 cells with rapamycin (Rap, an mTORC1 inhibitor), dorsomorphin (Dor, an AMPK inhibitor), and PF-06409577 (PF, an AMPK agonist). No cytotoxicity was observed in Rap-, Dor-, and PF-treated PK-15 cells (Fig. 5F through H). For control cells, inhibiting mTORC1 with Rap or AMPK with Dor noticeably reduced PRV proliferation while activating AMPK with PF enhanced it (Fig. 5I). Interestingly, in shVDR-2 PK-15 cells, Rap and Dor further decreased PRV proliferation, whereas PF brought the viral titer back to levels comparable with untreated control cells (Fig. 5I). Collectively, these findings demonstrate that VDR-mediated Ca^2+^ absorption is essential for activating the PI3K/AKT/mTORC1 and AMPK/mTORC1 pathways, ultimately facilitating PRV proliferation.

### VDR and the PI3K/AKT/mTORC1 and AMPK/mTORC1 pathways are involved in PRV-induced lipid synthesis

**Fig 6:**
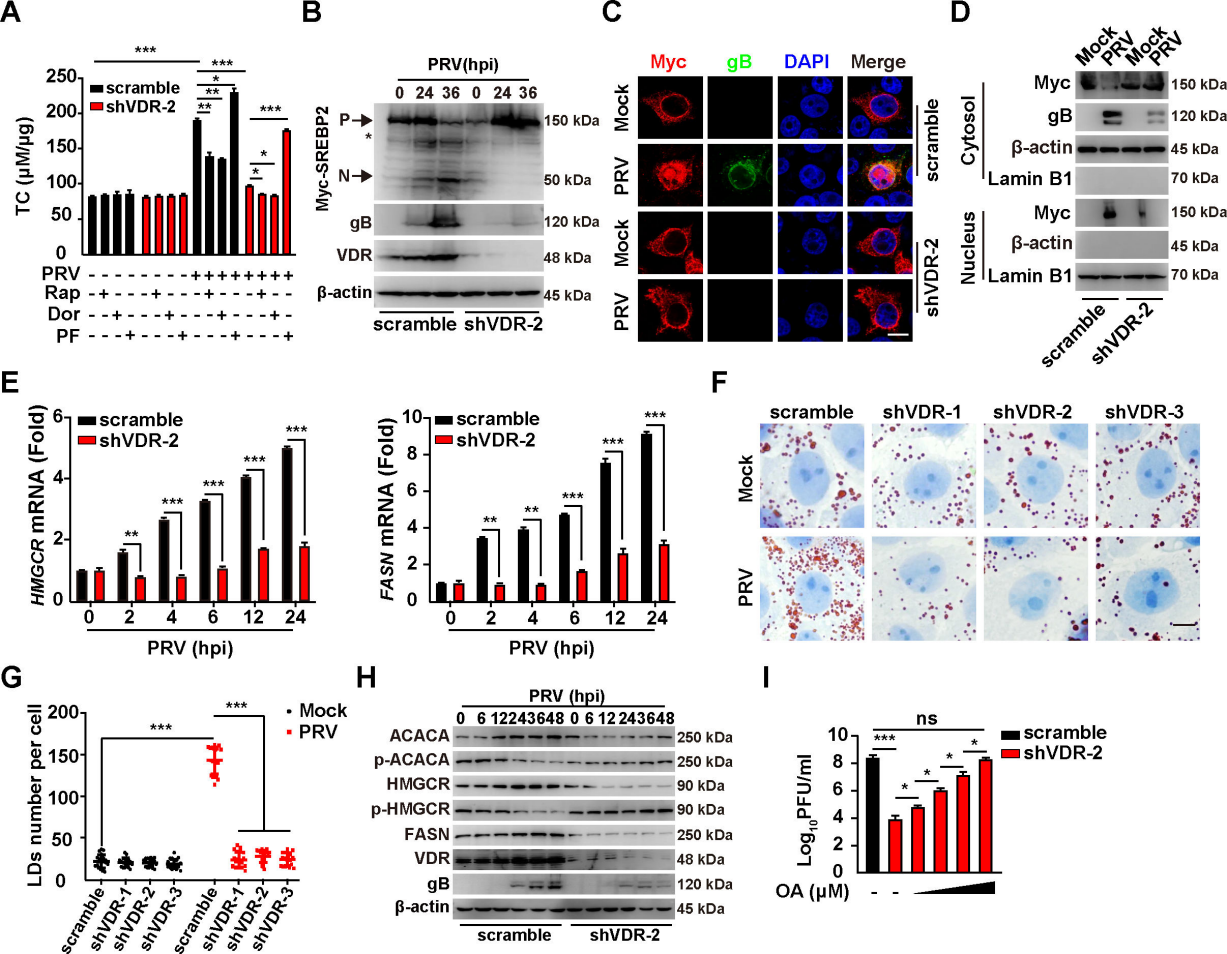
PRV induces lipid synthesis through the AMPK-mTORC1 pathway. (**A**) Scramble and shVDR-2 PK15 cells were treated with Rap (10 µM), Dor (100 nM), and PF (10 µM) as indicated and mock-infected or infected with PRV-QXX (MOI = 1) for 24 h. Intracellular TC levels were analyzed by a cholesterol assay kit. ^*^P < 0.05, ^**^P < 0.01, ^***^P < 0.001. (**B**) Scramble and shVDR-2 PK15 cells were transfected with Myc-SREBP2 for 12 h and then infected with PRV-QXX (MOI = 0.1) for 0–36 h. The protein levels of Myc-SREBP2, PRV gB, VDR, and β-actin were analyzed by immunoblotting analysis. P, precursor of Myc-SREBP2; N, nuclear form of Myc-SREBP2. The asterisk indicated the non-specific band. (**C**) Scramble and shVDR-2 PK15 cells were transfected with Myc-SREBP2 for 12 h and then mock-infected or infected with PRV-QXX (MOI = 1) for 24 h. The subcellular localization of Myc-SREBP2 was detected by fluorescence analysis. Scale bar, 10 µm. (**D**) Scramble and shVDR-2 PK15 cells were transfected with Myc-SREBP2 for 12 h and then mock-infected or infected with PRV-QXX (MOI = 1) for 24 h. Myc-SREBP2 was detected with immunoblotting analysis in the cytosol and nuclear fraction. β-actin (indicating cytosol) and Lamin B1 (indicating nucleus) served as loading controls. (**E**) Scramble and shVDR-2 PK15 cells were infected with PRV-QXX (MOI = 1) for 0–24 h. The mRNA levels of HMGCR and FASN were analyzed using qRT-PCR analysis. ^**^P < 0.01, ^***^P < 0.001. (**F**) Scramble, shVDR-1, shVDR-2, and shVDR-3 PK15 cells were mock-infected or infected with PRV-QXX (MOI = 1) for 24 h. LDs were detected by oil red O staining. Scale bar, 10 µm. (**G**) Quantification of the number of LDs per cell from (**E**) by ImageJ (*n* = 20). ^***^P < 0.001. (**H**) Scramble and shVDR-2 PK15 cells were infected with PRV-QXX (MOI = 0.1) for 0–48 h. The protein levels of ACACA, p-ACACA, HMGCR, p-HMGCR, FASN, VDR, PRV gB, and β-actin were analyzed by immunoblotting analysis. (**I**) Scramble and shVDR-2 PK15 cells were treated with OA (0, 10, 50, 100, and 150 µM) as indicated, and simultaneously infected with PRV-QXX (MOI = 1) for 24 h. The viral titer was analyzed by a PFU assay. ^*^P < 0.05, ^***^P < 0.001. ns, no significance.

The PI3K/AKT/mTORC1 and AMPK/mTORC1 pathways are implicated in the regulation of lipid synthesis (21, 22). Upon assessing total cellular cholesterol (TC), we observed a reduction in TC levels in PRV-infected shVDR-2 PK-15 cells compared with uninfected controls (Fig. 6A). Treatment with Rap and Dor led to a decrease in TC levels, whereas PF administration resulted in an increase (Fig. 6A). These results indicate that the PI3K/AKT/mTORC1 and AMPK/mTORC1 signaling pathways are crucial in regulating lipid synthesis induced by PRV infection.

To further understand the role of VDR in this context, we explored its effect on lipid synthesis during PRV infection. Sterol regulatory element-binding proteins (SREBPs) are key transcription factors in the control of lipid synthesis (23). Given PRV’s stimulation of lipid synthesis for optimal replication (24, 25), we analyzed the activation of SREBP2. After transfecting a plasmid encoding Myc-SREBP2 into control and shVDR-2 PK-15 cells followed by PRV-QXX infection, the analysis through immunoblotting analysis showed that the nuclear form of Myc-SREBP2 was present in PRV-infected control cells but absent in PRV-infected shVDR-2 PK-15 cells (Fig. 6B). Moreover, immunofluorescence analysis revealed that PRV infection induced the nuclear translocation of Myc-SREBP2, a process that was disrupted by VDR knockdown (Fig. 6C). This finding was further verified by a subcellular fractionation assay (Fig. 6D). We also measured the transcription levels of key enzymes in lipid synthesis, 3-hydroxy-3-methylglutaryl-CoA reductase (HMGCR) and fatty acid synthase (FASN), both regulated by SREBP, which were found to be elevated following PRV infection (Fig. 6E). In contrast, in VDR-knockdown PK-15 cells, PRV failed to enhance HMGCR and FASN transcription (Fig. 6E). These findings suggest that VDR is essential for the activation of SREBP induced by PRV.

Oil red O staining highlighted a significant decrease in lipid droplets (LDs) in VDR-silenced cells post-PRV infection (Fig. 6F and G). Immunoblotting analysis further showed an upregulation of ACACA, HMGCR, and FASN in control but not in shVDR-2 PK-15 cells following PRV infection (Fig. 6H). Increased phosphorylation of HMGCR and ACACA was observed in VDR-deficient cells, suggesting potential inactivation through VDR silencing—a process known to enzymatically deactivate these enzymes upon phosphorylation (Fig. 6H) (26). The addition of oleic acid (OA) to shVDR-2 PK-15 cells was capable of restoring PRV progeny production to levels similar to those in untreated control cells (Fig. 6I). Collectively, these findings suggest that VDR-mediated activation of PI3K/AKT/mTORC1 and AMPK/mTORC1 pathways plays a critical role in PRV-induced lipid synthesis.

### VDR is involved in PRV entry, assembly, and release

**Fig 7:**
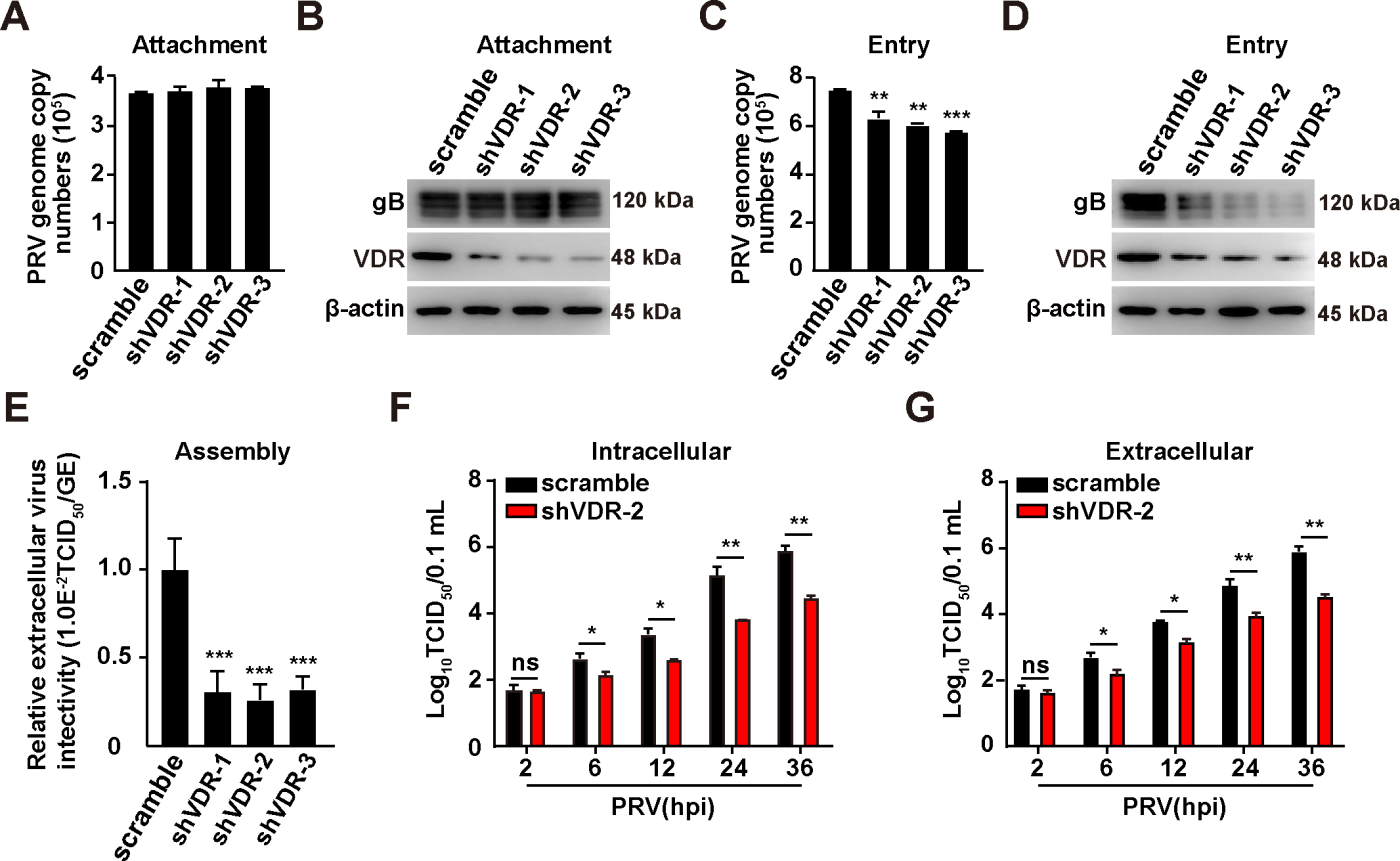
Knockdown of VDR affects PRV entry, assembly, and release. (**A**) Scramble, shVDR-1, shVDR-2, and shVDR-3 PK-15 cells were incubated with PRV (MOI = 10) at 4°C for 2 h. After the cells were washed three times with ice-cold PBS, viral attachment was assessed by qRT-PCR analysis of genome copy numbers on the PM. (**B**) Scramble, shVDR-1, shVDR-2, and shVDR-3 PK-15 cells were incubated with PRV (MOI = 10) at 4°C for 2 h. After the cells were washed three times with ice-cold PBS, viral attachment was assessed by immunoblotting analysis of gB expression. (**C**) Scramble, shVDR-1, shVDR-2, and shVDR-3 PK-15 cells were incubated with PRV (MOI = 10) at 4°C for 2 h, extensively washed with ice-cold PBS three times, and then incubated at 37°C for 10 min to allow entry. After washing with trypsin (1 mg/mL) to remove residual virions on the PM, viral entry was detected by qRT-PCR analysis of viral genome copy numbers in the cells. **P < 0.01, ***P < 0.001. (**D**) Scramble, shVDR-1, shVDR-2, and shVDR-3 PK-15 cells were incubated with PRV (MOI = 10) at 4°C for 2 h, extensively washed with ice-cold PBS three times, and then incubated at 37°C for 10 min to allow entry. After washing with trypsin (1 mg/mL) to remove residual virions on the PM, viral entry was detected by immunoblotting analysis of gB expression. (**E**) Scramble, shVDR-1, shVDR-2, and shVDR-3 PK-15 cells were infected with PRV (MOI = 5) for 24 h. The efficiency of viral assembly in the supernatants was determined by comparing the infectious titers (TCID_50_ per milliliter) with the total PRV genome equivalents (GE). ***P < 0.001. (**F and G**) Scramble and shVDR-2 PK-15 cells infected with PRV-QXX (MOI = 5) had their extracellular (**F**) and intracellular (**G**) viruses harvested and subjected to a TCID_50_ assay to determine viral titers from 2–36 hpi. *P < 0.05, **P < 0.01. ns, no significance.

To further investigate how VDR influenced viral infection, scrambled and shVDR-2 PK-15 cells were incubated with PRV at 4°C for 2 h. The viral attachment assay indicated that VDR knockdown did not disrupt viral attachment, as evidenced by qRT-PCR results measuring the viral genome copy number and by immunoblotting analysis of gB on the plasma membrane (PM) (Fig. 7A and B). Additionally, viral entry was assessed using qRT-PCR and immunoblotting analysis. The qRT-PCR analysis revealed that the incoming viral genome copy number, as well as immunoblotting analysis of gB expression, were lower in VDR-knockdown cells compared with control cells (Fig. 7C and D). Following that, we examined the impact of VDR knockdown on viral assembly and release, determining that suppressing VDR expression impeded the infectivity of PRV assembly and the resulting virus progeny (Fig. 7E through G). Overall, these results indicate that VDR is involved in PRV entry, assembly, and release.

## DISCUSSION

**Fig 8:**
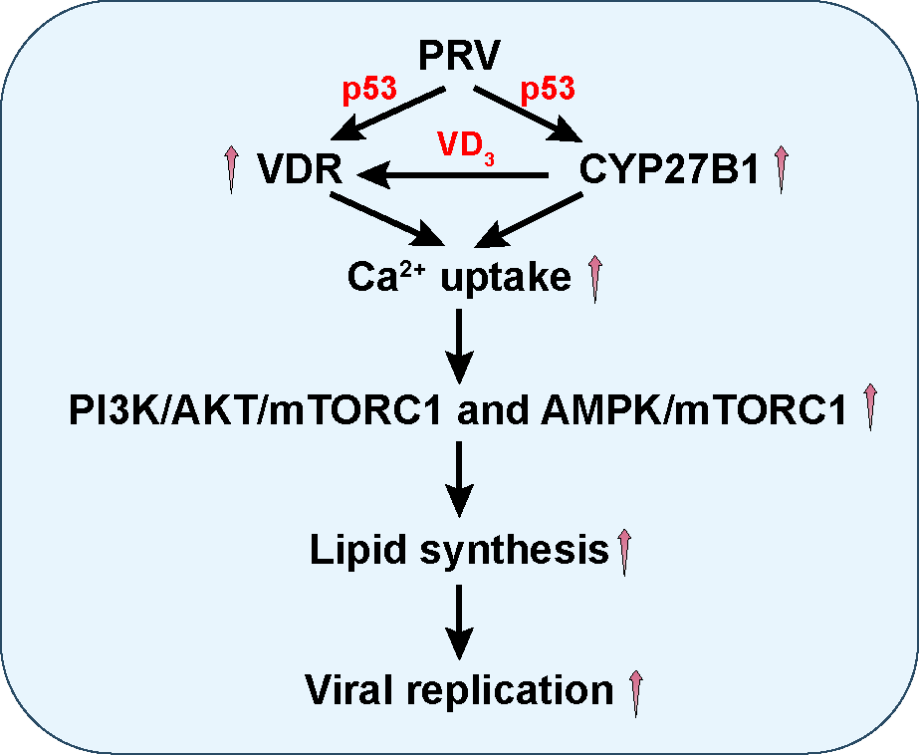
A schematic model showing PRV infection promotes VDR expression, thereby enhancing Ca^2+^ absorption and activating PI3K/AKT/mTORC1- and AMPK/mTORC1-mediated lipid synthesis.

Although previous investigations have delved into the relationship between VDR and viral infection, the specific interaction between PRV and VDR remains underexplored. In our study, we provide new insights into how PRV infection triggers an influx of extracellular Ca^2+^ via VD_3_/VDR signaling pathway. This influx, in turn, activates lipid synthesis pathways, specifically the AMPK/mTORC1 and PI3K/AKT/mTORC1 pathways, facilitating the replication of PRV (Fig. 8). Notably, our findings suggest that the VD_3_/VDR signaling pathway plays a crucial role in modulating the cellular environment to promote viral replication, underscoring a complex interplay between host cellular processes and viral life cycles.

It has been revealed that across three PRV-strain-infected cell types, lipids and lipid-like molecules comprise more than 50% of the altered metabolites (27). The majority of these differentially expressed metabolites, influenced by PRV infection, are associated with glycerophospholipid metabolism, sphingolipid metabolism, as well as purine and pyrimidine metabolism, with lipid metabolites representing the largest proportion (28). 25-Hydroxyvitamin D impairs SREBP activation by inducing proteolytic processing and ubiquitin-mediated degradation of SREBP cleavage-activating protein, thereby decreasing SREBP levels and lipid synthesis independent of VDR (29). Additionally, VDR has been found to influence a range of functions in cells infected with various herpesviruses. For instance, VDR acts as a binding partner of Epstein-Barr virus EBNA-3, which, upon binding to VDR, hinders the activation of VDR-dependent genes and shields LCLs from vitamin-D3-induced growth arrest and/or apoptosis (30). Within hours of lytic infection, HCMV becomes resistant to vitamin D therapy *in vitro* by downregulating VDR (31). Additionally, we report that PRV employs VDR-mediated lipid synthesis for PRV proliferation. All the findings suggest that lipid metabolism is critical role in PRV proliferation.

Lipid metabolism is crucial throughout the replication cycle of various viruses (32). Our findings suggest that VDR-mediated lipid metabolism plays a significant role in PRV entry. Previous investigations have shown that deficiencies in Niemann-Pick C1 markedly inhibit PRV proliferation by reducing cholesterol levels in the plasma membrane. This reduction impairs the dynamics of clathrin-coated pits (CCPs), which are essential for clathrin-mediated endocytosis involved in PRV entry (33). Additionally, the activation of liver X receptors, leading to decreased cellular cholesterol, has been identified as critically affecting the dynamics essential for PRV entry through CCPs (24). Furthermore, cholesterol-enriched lipid rafts in the host cell membrane are instrumental in the entry of herpes simplex virus type 1 (HSV-1) by aiding the fusion process (34). Consequently, we hypothesize that VDR-mediated lipid regulation is crucial for PRV entry due to its impact on lipid rafts and CCPs.

We also propose that VDR-mediated lipid metabolism contributes to PRV assembly and release. The acquisition of a lipid envelope from host cell membranes, a key phase in the replication cycle of herpesviruses, involves the viral capsid envelopment. Human cytomegalovirus, for instance, employs its viral proteins to modify the lipid composition at its assembly sites, concentrating glycosphingolipids and cholesterol to enhance viral envelopment (35). Similarly, varicella-zoster virus induces changes in host lipid metabolism, thus supporting viral envelopment and initial virion assembly steps (36). Sphingolipids and cholesterol, fundamental components of lipid rafts, are essential for the assembly and budding of numerous herpesviruses. For example, alterations in host cell lipid metabolism, notably the sphingomyelin biosynthesis pathway, influence the envelopment and egress of Epstein-Barr virus (37). Additionally, the exploitation of phospholipase D, which alters host cell phospholipids, has been observed in HSV to optimize viral envelopment and release (38). Thus, PRV may utilize similar mechanisms mediated by VDR to facilitate its assembly and release.

The function of VDR in Ca^2+^ absorption is pivotal for both intestinal health and skeletal integrity (18, 39, 40). Children with inactivating mutations in the VDR gene, such as those seen in type II genetic rickets, exhibit disruptions in Ca^2+^ metabolism, notably characterized by reduced efficiency of intestinal Ca^2+^ absorption (41). Notably, the altered structure of growth plates stands as a significant clinical, radiological, and histological indicator of rickets. This condition is observable in both humans and mice experiencing either vitamin D deficiency or the inactivation of VDR or CYP27B1 (42, 43). Our current study reveals that VDR plays a crucial role in facilitating Ca^2+^ absorption in porcine kidney epithelial PK-15 cells during PRV infection, thereby broadening our understanding of VDR’s significance in the context of infectious diseases. Moreover, our observations suggest that PRV infection leads to the activation of p53, which in turn enhances VDR expression. Xun Li and colleagues have documented that p53 positively influences both the replication and pathogenesis of PRV *in vitro* and *in vivo* (44). Our findings elucidate a fundamental mechanism by which p53 promotes PRV replication, offering deeper insights into the intricate interplay between host cellular pathways and viral proliferation.

We also demonstrate that p53 plays a crucial role in upregulating the expression of CYP27B1, consequently enhancing VD_3_ synthesis. No studies have directly linked the regulation of CYP27B1 expression to p53. Additionally, our findings suggest that in the absence of VDR, VD_3_ is unable to promote PRV proliferation, highlighting the essential role of the VD_3_-VDR interaction in this process. VD_3_ activates the transcriptional activity of VDR (45), leading to increased Ca^2+^ absorption in the intestine, enhanced Ca^2+^ reabsorption in the kidneys, and augmented Ca^2+^ resorption in bones (46, 47). Furthermore, VD_3_ can instigate a rapid influx of Ca^2+^ from the extracellular space through voltage-independent channels in rat osteosarcoma cells (48), trigger the release of Ca^2+^ from intracellular stores in osteoblasts (46), and activate protein kinase pathways, which may be either Ca^2+^-dependent or -independent (46, 47). It is of interest to identify the specific voltage-independent channel facilitating VDR-dependent Ca^2+^ absorption and determine whether it is transcriptionally regulated by the VDR.

Moreover, we uncover that VDR-mediated Ca^2+^ absorption during PRV infection relies on the activation of the PI3K/AKT/mTORC1 and AMPK/mTORC1 pathways. mTORC1 and AMPK are critical for the regulation of cellular nutrition and metabolism, interacting through several distinct mechanisms (49–51). For instance, Porcine circovirus type 2 has been shown to activate CaMKII by elevating intracellular Ca^2+^ levels, where activated CaMKII can subsequently engage AMPK and CaMKI, thereby employing autophagy to bolster its replication (52). This study reveals that VDR can regulate mTORC1 and AMPK during PRV infection. The inhibition of VDR amidst PRV infection disrupts intracellular Ca^2+^ homeostasis, which suppresses AMPK and mTORC1, leading to an inhibition of lipid synthesis.

## MATERIALS AND METHODS

### Mice

The *p53^+/+^* and *p53^˗/˗^* (B6.129S2-*Trp53^tm1Tyj/J^*) mice on a C57BL/6J background were purchased from the Jackson Laboratory. Genotyping was performed by PCR according to the manufacturer’s protocol. Female C57BL/6 J mice, aged 6–8 weeks, were used for this study. Mice were housed in specific pathogen-free facilities under a 12 h light-dark cycle and at a controlled temperature of 22°C. Animal protocols were carried out in compliance with the Guide for the Care and Use of Laboratory Animals and the applicable ethical regulations of Henan Agricultural University

### Cells and viruses

PK-15, 3D4/21 and HEK293T cells were maintained in DMEM supplemented with 10% FBS, 1% penicillin/streptomycin, and 1% glutamine, at 37°C and 5% CO_2_. For the generation of shRNA knockdown cells, HEK293T cells were co-transfected with either scrambled shRNA (5′-GCCACAACGTCTATATCATGG-3′) or target gene-specific shRNA sequences (shVDR-1: 5′-GCTTCCATTTCAATGCTATGA-3′; shVDR-2: 5′-GGCTTCCATTTCAATGCTATG-3′; shVDR-3: 5′-GCCACCGGCTTCCATTTCAAT-3′; shCYP27B1-1: 5′-GGACCAGATGTTTGCATTTGC-3′; shCYP27B1-2: 5′-GGTCACTCTGTGTCACTATGC-3′; shCYP27B1-3: 5′-GCAGAGCTTGAGCTGCAAATG-3′), along with a packaging plasmid (psPAX2) and envelope plasmid (pMD2.G). The medium was changed 6 h later, and viral particles were harvested after an additional 48 h, then used to infect parental cells, which were subsequently selected with puromycin. The virulent PRV isolate QXX (PRV-QXX) was kindly donated by Yong-Tao Li from the College of Veterinary Medicine, Henan Agricultural University (53).

### Chemicals and antibodies

1,25-Dihydroxyvitamin D3 (HY-10002), pifithrin-β (HY-16702A), BAPTA-AM (HY-100545), PF-06409577 (HY-103683), dorsomorphin (HY-13418A), rapamycin (HY-10219), and oleic acid (HY-N1446) were purchased from MedChemExpress. 1,25-Dihydroxyvitamin D3 ELISA kit (D751006) was purchased from Sangon Biotech (Shanghai). BODIPY 493/503 (D3922) was purchased from Invitrogen. Oil red O (O0625) was purchased from Sigma-Aldrich.

Anti-p53 (10442–1-AP), anti-mTORC1 (66888–1-Ig), anti-p-mTORC1 (67778–1-Ig), anti-AKT (60203–2-Ig), anti-p-AKT (66444–1-Ig), anti-β-actin (66009–1-Ig), anti-Myc (16286–1-AP), anti-EGFP (50430–2-AP), and anti-PI3K (67071–1-Ig) antibodies were purchased from Proteintech. Anti-VDR (12550), anti-p-p53 (9284), anti-p-AMPKα (50081), anti-AMPKα (2532), anti-p-PI3K (17366), anti-ACACA (3676), and anti-p-ACACA (11818) antibodies were purchased from Cell Signaling Technology. Anti-FASN (ab22759) and anti-CYP27B1 (ab206655) antibodies were purchased from Abcam. The anti-HMGCR (MABS1233) antibody was purchased from Sigma-Aldrich. The anti-p-HMGCR (bs-4063R) antibody was purchased from Bioss. Mouse monoclonal antibody against PRV VP16 was a gift from Jing-Fei Wang (Harbin Veterinary Research Institute, Harbin, China). Antisera against PRV UL35 was generated by immunizing mice with purified recombinant UL35. The anti-PRV gB antibody was used as previously described. (25).

### Plasmid construction and transfection

The coding sequences of VDR were amplified from the cDNA of PK-15 cells and cloned into pEGFP-C1 to generate EGFP-VDR. For the luciferase reporter assay, the porcine VDR (Gene ID: 396628) promoter of 1,800 bp upstream of the transcription initiation site (+1) and p53 binding site mutants were synthesized by Genscript and cloned into pGL3-Basic (Promega, E1751) using *Kpn*I and *Hind*III to generate VDR-Luc plasmids. The p53 binding site 1 (-135) sequence 5′-AGACATG-3′ was mutated to 5′-CACACCA-3′ (VDR-Luc mut1). The p53 binding site 2 (-871) sequence 5′-GCATGCTT-3′ was mutated to 5′-AACCAACC-3′ (VDR-Luc mut2). The p53 binding site 3 (-1254) sequence 5′-ACAAGTCC-3′ was mutated to 5′-CACCACAA-3′ (VDR-Luc mut3). Transfection with these expression plasmids was performed in PK-15 cells using TurboFect (Thermo Fisher Scientific, R0531), according to the manufacturer’s instructions.

### Cell viability analysis

Cell viability was determined using the CCK-8 assay. Briefly, cells were seeded into 96-well plates at the indicated doses for 0–48 h. Ten microliters of CCK-8 solution (Biodragon, BDXB0002) were added to each well. After 2 h of incubation, the optical density of each well was measured at a wavelength of 450 nm.

### ChIP assay

PK-15 cells were cross-linked with DMEM containing 1% formaldehyde for 15 min and then incubated in 125 mM glycine for 5 min. After being washed with PBS three times, the cells were incubated in lysis buffer (10 mM Tris-HCl, pH 7.5, 10 mM KCl, 5 mM MgCl_2_, and 0.5% NP40) supplemented with a protease inhibitor cocktail (MedChemExpress, HY-K0010) on ice for 10 min and centrifuged at 400 × *g* for 5 min. The cell pellets containing chromatin were resuspended in SDS lysis buffer (50 mM Tris-HCl, pH 7.9, 10 mM EDTA, and 0.5% SDS) supplemented with a protease inhibitor cocktail (MedChemExpress) and sonicated into fragments with an average length of 1 kb. ChIP assays were performed with IgG (Proteintech, B900620) or an antibody against p53. The specific primers used to amplify the VDR promoter were as follows: Fw: 5ʹ-TTTCTGCACTCTCCCAC-3ʹ and Rv: 5ʹ-GGCGCGACTGAGTCTG-3ʹ.

### RNA-seq

RNA was extracted using TRIzol reagent (TaKaRa, 9108) according to the manufacturer’s protocol, with *n* = 3 biologically independent repeats per group. RNA-seq libraries were constructed using the TruSeq Stranded Total RNA Library Prep Kit (Illumina, RS-122–2503) according to the manufacturer’s instructions. The libraries were sequenced on the Illumina NovaSeq 6000 platform, and 150 bp paired-end reads were generated. Raw reads in fastq format were first processed with Fastp, and low-quality reads were removed to obtain clean reads. Differential expression analysis was performed using DESeq2.

### Immunoblotting analysis

Cells were harvested and lysed using lysis buffer (50 mM Tris–HCl, pH 8.0, 150 mM NaCl, 1% Triton X-100, 1% sodium deoxycholate, 0.1% SDS, and 2 mM MgCl_2_) supplemented with a protease inhibitor cocktail (MedChemExpress). Protein samples were separated by SDS-PAGE and then transferred to a membrane. This membrane was incubated in 5% nonfat milk for 1 h at room temperature. Subsequently, the membrane was incubated with primary antibodies overnight at 4°C, followed by incubation with horseradish peroxidase-conjugated secondary antibodies for 1 h at room temperature. Immunoblotting results were visualized using Luminata Crescendo Western HRP substrate (Millipore, WBLUR0500) on a GE AI600 imaging system.

### RNA extraction and qRT-PCR

Total RNA was isolated using TRIzol reagent (TaKaRa, 9108) and subjected to cDNA synthesis using a PrimeScript RT reagent kit (TaKaRa, RR047A). qRT-PCR was performed in triplicate using SYBR Premix Ex Taq (TaKaRa, RR820A), according to the manufacturer’s instructions, and data were normalized to the level of *ACTB* expression in each individual sample. Melting curve analysis indicated the formation of a single product in all cases. The 2^˗ΔΔCt^ method was used to calculate relative expression changes. Primers used for qRT-PCR are follows: porcine *ACTB*-Fw: 5′-CTGAACCCCAAAGCCAACCGT-3′, porcine *ACTB*-Rv: 5′-TTCTCCTTGATGTCCCGCACG-3′; porcine *VDR*-Fw: 5′-CCGGACCAGAGTCCTTTTGG-3′, porcine *VDR*-Rw: 5′-GGGGTCAGGTAAGGAAGTGC-3′; porcine *VEGFC*-Fw: 5′-CTGTGAGGCTCTCCCCTGA-3′, porcine *VEGFC*-Rw: 5′-CAGCAAGTGCATGGTGGAC-3′; porcine *NOS2*-Fw: 5′-GCCCAGAGGGCTTTATCACTG-3′, porcine *NOS2*-Rw: 5′-GATTTCTTTGCTGTCTCCGCC-3′; porcine *ATP2A1*-Fw: 5′-CCCTCTGCCGATGATCTTCAA-3′, porcine *ATP2A1*-Rw: 5′-CACGGTTCAAAGAGGTGGAG-3′; porcine *PDE1A*-Fw: 5′-GCAGAGCAGTTTGTGTGCAG-3′, porcine *PDE1A*-Rw: 5′-GTATGCCTTTCAGACGTTGCC-3′; porcine *PLCD3*-Fw: 5′-GCGTTGAAGAAGATGGGCCT-3′, porcine *PLCD3*-Rw: 5′-AGAAGATGTGCTGAGACGGC-3′; porcine *SLC8A2*-Fw: 5′-GAGGTGCCATTTCCCCTCCAT-3′, porcine *SLC8A2*-Rw: 5′-GAGGAAGTATTGGCCAGGGATT-3′; porcine *P2R × 2*-Fw: 5′-AAGGTGATCGTGGTACGTGT-3′, porcine *P2R × 2*-Rw: 5′-CCTTGGTGATGACGGAGCTT-3′; porcine *CACNA1S*-Fw: 5′-GGCTACTTTGGAGACCCCTG-3′, porcine *CACNA1S*-Rw: 5′-CTGGAGGCCAGGAAAGTGTC-3′; porcine *GRM5*-Fw: 5′-CGAGGAGCGAGTGGCTG-3′, porcine *GRM5*-Rw: 5′-ACCACCATGTCCCCAGGTAT-3′; and porcine *FGFR1*-Fw: 5′-TCACCAACGAGGATCAAGCC-3′, porcine *FGFR1-*Rw: 5′-TGCGGTTAGAGGTTGGTGAC-3′.

### Immunofluorescence analysis

Cells cultured on coverslips in 24-well plates were fixed with 4% (wt/vol) paraformaldehyde at room temperature for 20 min. After washing with PBS three times, the cells were permeabilized with 0.1% Triton X-100 for 10 min, and then blocked with 10% FBS. The specific primary antibodies, diluted in 10% FBS, were added to the cells and incubated for 1 h at room temperature. After washing with PBS three times, the cells were incubated with the appropriate secondary antibodies diluted in 10% FBS for 1 h at room temperature. The nuclei were stained with DAPI for 10 min at room temperature, mounted with FluorSave Reagent (Millipore, 345789), and examined on a Zeiss LSM 800 confocal microscope.

### Ca^2+^ measurements

Cytosolic Ca^2+^ was measured as previously described, with minor modifications (54). A Fluo-4 Calcium Assay Kit (Beyotime, S1061S) was used to measure cytoplasmic Ca2+. Briefly, at the indicated time points, 100 µL of Fluo-4 dye-loading solution was aliquoted into wells of a 24-well plate containing 1 × 10^5^ cells per well and incubated for 30 min at 37°C, followed by another 30 min at room temperature. Ca^2+^ flux was determined by measuring fluorescence at Ex/Em = 490/525 nm.

To visualize the correlation between PRV infection and cytoplasmic Ca^2+^, the cells were infected with PRV, and the Ca^2+^ concentration was measured by Fluo-4 (Beyotime). Briefly, scramble and shVDR-2 PK-15 cells were infected with PRV at the indicated MOI or time points, and then the cells were incubated with Fluo-4 at a volume of 250 µL. After incubation at 37°C in a dark incubator for 30 minutes, the cells were observed with a fluorescence microscope.

### Lipid quantification

TC was measured with a cholesterol assay kit (Solarbio, BC1985) according to the manufacturer’s instructions. The values were normalized to the total cellular protein content.

### Plaque assay

Vero cells were cultured to confluency in six-well plates and inoculated with serially diluted viruses (10^−1^-fold 10^−7^-fold) for 1 h at 37°C. The excess viral inoculum was removed by washing with PBS. Next, 4 mL of DMEM/1% methylcellulose was added to each well, and the cells were further cultured for 4–5 days. The cells were fixed with 4% paraformaldehyde for 15 min and then stained with 1% crystal violet for 30 min before the plaques were counted.

### Statistics

All data were analyzed using GraphPad Prism 8 software and a two-tailed Student’s *t*-test. *P* < 0.05 was considered statistically significant. Data are presented as the mean ± standard deviation from three independent experiments.
